# Social determinants of health and self-rated health status: A comparison between women with HIV and women without HIV from the general population in Canada

**DOI:** 10.1371/journal.pone.0213901

**Published:** 2019-03-21

**Authors:** Mostafa Shokoohi, Greta R. Bauer, Angela Kaida, Ashley Lacombe-Duncan, Mina Kazemi, Brenda Gagnier, Alexandra de Pokomandy, Mona Loutfy, On Behalf of the CHIWOS Research Team

**Affiliations:** 1 Epidemiology & Biostatistics, Schulich School of Medicine & Dentistry, The University of Western Ontario, London, ON, Canada; 2 Faculty of Health Sciences, Simon Fraser University, Burnaby, British Columbia, Canada; 3 Factor-Inwentash Faculty of Social Work, University of Toronto, Toronto, Ontario, Canada; 4 School of Social Work, University of Michigan, Ann Arbor, MI, United States of America; 5 Women’s College Research Institute, Women’s College Hospital, Toronto, Ontario, Canada; 6 Department of Family Medicine, McGill University, Montreal, Quebec, Canada; 7 McGill University Health Centre, Montreal, Quebec, Canada; 8 Faculty of Medicine, University of Toronto, Toronto, Ontario, Canada; 9 Dalla Lana School of Public Health, University of Toronto, Toronto, Ontario, Canada; The University of Warwick, UNITED KINGDOM

## Abstract

**Background:**

Women living with HIV (WLWH) continue to experience poorer outcomes across the HIV care cascade and overall health, an appreciable proportion of which may not be disease-related but due to socio-structural barriers that impact health. We compared socio-structural determinants of health and self-rated health between WLWH and expected general population values.

**Methods:**

Prevalences of socio-structural determinants and self-rated health were estimated from 1,422 WLWH aged 16+ in the 2013–2015 Canadian HIV Women’s Sexual and Reproductive Health Cohort Study (CHIWOS). Prevalences were also estimated from 46,831 general population women (assumed HIV-negative) in the 2013–2014 Canadian Community Health Survey (CCHS), standardized to the age/ethnoracial group distribution of WLWH. Standardized prevalence differences (SPDs) and 95% confidence intervals (CI) were reported.

**Results:**

Compared to general population women, a higher proportion of WLWH reported annual personal income <$20,000 (SPD 42.2%; 95% CI: 39.1, 45.2), indicating that 42.2% of WLWH experienced this low income, in excess of what would be expected of Canadian women of similar ages/ethnoracial backgrounds. A higher proportion of WLWH reported severe food insecurity (SPD 43.9%; 40.2, 47.5), poor perceived social support (SPD 27.4%; 22.2, 33.0), frequent racial (SPD 36.8%; 31.9, 41.8) and gender (SPD 46.0%; 42.6, 51.6) discrimination, and poor/fair self-rated health (SPD 12.2%; 9.4, 15.0).

**Conclusions:**

Significant socio-structural inequalities and lower self-rated health were found among WLWH compared to general population women. Such inequities support the integration of a social-determinants approach, social service delivery, and programming into HIV care, with additional resource allocation tailored to the particular needs of WLWH.

## Introduction

Research has shown substantial improvements in health outcomes of people living with HIV (PLWH) since the introduction of combination antiretroviral therapy (cART); for example, life expectancy for those who receive cART has been approaching that of the general population [1, 2]. Despite the remarkable successes achieved in HIV outcomes, they are still not ideal, particularly among women living with HIV (WLWH). A recent Canadian study demonstrated that reductions in health-adjusted life expectancy among those living with HIV were larger for women than men [2]. In addition, Canadian studies have documented that a higher proportion of women experience poorer “quality of care” in Canada, indicating the existence of gender inequities in access and adherence to HIV treatment even in a universal healthcare system [3, 4].

Although HIV is now widely known as a chronic but manageable illness where appropriate care and treatment services are accessible [5], multiple interpersonal and structural factors–situated within social determinants of health (SDoH), continue to dampen HIV care and treatment efforts. The World Health Organization (WHO) defines the SDoH as “the conditions in which people are born, grow, live, work, and age.”[6] Literature has uncovered the contribution of these socio-structural disadvantages in shaping the HIV epidemic among PLWH [7–9].

In turn, living with HIV can also cause greater vulnerability to socio-structural disadvantages; for example, PLWH experience food insecurity even after an HIV diagnosis, and employment loss, particularly among women [10, 11]. Despite advances in HIV interventions, PLWH continue to experience challenges to maintaining their health due to the barriers linked with SDoH [8, 9, 12]. For example, socioeconomic inequities, housing instability, food insecurity, HIV-related stigma, and discrimination have been correlated with poorer HIV care, treatment responses, and clinical outcomes [10, 12–17]. WLWH are a population that face relatively lower socioeconomic status, and broader, systemic inequities that impact their health and wellbeing [3, 4].

In Canada, women now represent nearly one-quarter of the estimated 75,500 PLWH [18]. WLWH in Canada are disproportionately from communities that experience marginalization. For example, 35.6% and 30.6% of new HIV diagnoses in women in 2014 were identified among Black and Indigenous women, respectively [18]. Canadian WLWH were shown having higher vulnerabilities to substance use, particularly cigarette smoking and illicit drug use, than Canadian women with a similar age/ethnoracial background [19]. Additional experiences of disadvantage, with regard to social determinants in particular, can result in poorer health outcomes, even in countries where cART is widely available [9]. However, the magnitude of inequalities in underlying socio-structural barriers among WLWH compared with the broader population have not yet been investigated as general population studies do not accurately identify HIV status, and HIV cohort studies often do not include enough women to ensure robust comparison to the broader population to assess differences. Understanding socio-structural barriers that WLWH face in excess of what would be expected is essential to minimize vulnerability to HIV, eliminate inequities in the HIV care cascade, reduce vulnerabilities to poor outcomes, and improve health and well-being.

Therefore, this study took advantage of comparable measures in two large data sets—CHIWOS for WLWH and the Canadian Community Health Survey for women of the general population—to investigate socio-structural determinants and self-rated health status among WLWH, and then compare them with the assumed HIV-negative general population of women, standardizing for age and ethnoracial variables.

## Methods

### Study cohorts

#### CHIWOS

We used the baseline survey (time-point 1) of the Canadian HIV Women’s Sexual and Reproductive Health Cohort Study (CHIWOS) of WLWH enrolled from 2013 to 2015 in Canada. As a community-based research study, CHIWOS applied the Greater Involvement of People Living with HIV/AIDS (GIPA) and Meaningful Involvement of Women Living with HIV/AIDS (MIWA) principles such that WLWH were integral to all steps of the research process [20, 21]. CHIWOS enrolled 1,422 WLWH aged ≥ 16, residing in British Columbia (BC), Ontario, and Quebec. Participants were recruited through peers, HIV clinics, AIDS Service Organizations, and online networks [20]. The survey was completed during an in-person interview at clinic or community sites or participants’ homes, or via phone/Skype if this was not possible. Information was collected using structured questionnaires, administered by trained Peer Research Associates (PRA) in English or French. Participants provided written or oral informed consent at enrolment. CHIWOS was approved by the Research Ethics Boards of Simon Fraser University, University of British Columbia/Providence Health, Women’s College Hospital and McGill University Health Centre. CHIWOS was approved by the Research Ethics Boards of Simon Fraser University, University of British Columbia/Providence Health, Women’s College Hospital and McGill University Health Centre.

#### CCHS

The Canadian Community Health Survey (CCHS) is a nation-wide population-based survey administered by Statistics Canada that collects self-reported data on various health-related information of approximately 65,000 Canadian residents annually.[22] Briefly, the CCHS uses a multistage, stratified cluster sampling design to target ~98% of Canadians aged ≥12 for inclusion in all provinces and territories. The CCHS excludes people living on reserves, full-time members of the Canadian Forces, the institutionalized residents, and residents of some remote areas. For the purpose of the present research, we used Statistics Canada’s Public Use Microdata Files to create a combined CCHS dataset within two years of 2013/2014. For consistency with CHIWOS, we limited the CCHS’s analytic sample to women aged ≥16 years old, residing in the three provinces (analytic sample = 46,851). To study day-to-day discrimination, we used the CCHS-Rapid Response on the Everyday Discrimination Scale (EDS) performed separately in 2013 (analytic sample = 6,936). CCHS collects data using both computer-assisted personal and telephone interviews. Statistics Canada’s Research Data Centre at the University of Western Ontario provided researchers of the current study with access to the CCHS microdata.

### Measures

The most widely used Canadian SDoH framework recognizes that the following socio-structural determinants can help elucidate existing health differences: Aboriginal status, disability, early life, education, employment and working conditions, food insecurity, health services, gender, housing, income and income distribution, race, social exclusion, social safety net, and unemployment and job security [23]. We chose only those measures whose content and/or wording were similar in the question stems allowing the measures comparable between the two surveys.

The following measures were compared: *relationship status* (single, living common-law or married, and separated/widowed/divorced), *education level* (below high school, completed high school, above high school to non-university degree, and obtained university degree), *yearly personal income* (<$20,000, $20,000 to $39,999, ≥ $40,000, and Not Stated), *yearly household income* (<$20,000, $20,000 to $39,999, and ≥ $40,000), and the *main source of income* (wages/salaries [paid jobs], employment insurance/compensation/welfare, others [e.g., Dividends and interest, Benefits from Pension Plan, no income, etc.], and don’t know/not stated).

CHIWOS examined *food sufficiency* and *food security* using Statistics Canada’s 4-item adult measure from the Household Food Security Survey Module [24]. The matched items were also found in CCHS. *Food sufficiency* was measured with a question about past-year household food sufficiency, with responses recoded into three categories: always had enough of the kinds of food they wanted to eat, had enough but not always the kinds of food they wanted to eat, and sometimes/often did not have enough to eat. Household *food security* over the last 12 months was measured by three items, “worried that food would run out,” “The food did not last, and there was no money to get more,” and “could not afford to eat balanced meals.” Binary response options for each item were created as 1 for “Sometimes/Often true” and 0 for “Never true.” We summed these three items to form a four-category ordinal measure: 0: food secure, 1: mildly food insecure, 2: moderately food insecure, and 3: severely food insecure. CCHS did not measure food security in BC; for comparability, we provided estimates for only Ontario and Quebec in CHIWOS.

*Perceived social support* was measured using a 4-item abbreviated version of the Medical Outcome Study Social Support Survey (MOS-SSS) [25], measuring four domains of emotional/informational, tangible, affectionate, and positive social interaction. Possible responses included strongly disagree (score 0), disagree, agree, and strongly agree (score 3) in CCHS and a five-point Likert scale, with responses recoded into four categories as none of the time (score 0), a little of the time, some or most of the time, and all of the time (score 3). Items were summed (range 0–12 points), with higher scores implying greater perceived social support. For the purpose of comparison, we created a binary measure with ≤6 indicating poorer social support. The analysis was limited to data from Quebec as CCHS did not measure social support in BC and Ontario.

*Racial discrimination* and *gender discrimination* measures were quantified using a modified version of the Everyday Discrimination Scale [26], with 5-item version in CCHS and 6-item version in CHIWOS. CCHS respondents were asked to specify how often they had experienced various forms of day-to-day mistreatments “because of your race” or “because of your gender.” Items included “You are treated with less courtesy or respect than other people,” “You receive poorer service than other people at restaurants or stores,” “People act as if they think you are not smart,” “People act as if they are afraid of you,” and “You are threatened or harassed.” CHIWOS asked the first question in two separate items, “You are treated with less courtesy,” and “You are treated with less respect.” The CCHS’s items were on a five-point scale (at least once a week, a few times a month, a few times a year, less than once a year, never), while they were on a six-point scale in CHIWOS (never, almost never, not that often, sometimes, frequently, almost every day). Two three-category measures were created for racial and gender discrimination, representing: never or almost never experienced any of the mistreatments, infrequent experience indicating less than once a year or not that often for any of the mistreatments, and frequent experience indicating more than once, or sometimes, or more in a year for any of the mistreatments.

*Self-rated health status* was measured in both surveys using a single question, “In general, would you say that your health is…?” We included an ordinal variable with five possible responses (excellent, very good, good, fair, and poor), and a binary recoded variable (poor/fair vs. good/very good/excellent).

### Statistical analyses

Proportions and associated 95% confidence intervals (CIs) for each measure were first estimated in CHIWOS (i.e., *observed* estimates). Then, the proportion of the same measure was estimated in the CCHS. Survey weights were incorporated into the analyses to account for the survey complexity and provide population-level estimates. The 95% CIs were constructed through the bootstrap variance estimation technique using a set of 500 replicates to account for the complex survey design effects [27]. Standardization method was used to account for the differences in population structure by age and ethnoracial group (**S1 Table**). These two variables are considered as the important confounders representing non-modifiable characteristics that differ between the study samples but are not a result of HIV status. To do this, we first produced a 16-category variable representing CHIWOS’s age and ethnoracial group structure (i.e., age with four categories: 16–35, 36–45, 46–55, or >55; and ethnoracial statues with four categories: white, African, Caribbean, Black (ACB), Indigenous, or other/multi-ethnicities. We applied CHIWOS’s combined age and ethnoracial distribution to the CCHS sample to make the two study populations of CHIWOS and CCHS identical with regard to the distribution of these two variables. After controlling the confounding impact of these two variables, we then provided the age-/ethnoracial-standardized estimates (i.e., *expected* estimates) of the SDoH measures and self-rated health. Standardization combines stratum-specific prevalence into a single summary estimate through taking a weighted average [28].

We reported the standardized prevalence differences (SPDs) to quantify the differences between the two study samples for each SDoH measure as well as self-rated health. The SPDs were calculated as the proportion of the observed estimates in CHIWOS minus the expected estimates from the CCHS adjusted for age/ethnoracial group identity; with the SPDs > 0 indicating a greater proportion of the given determinant among WLWH and can be interpreted as the proportion of WLWH experiencing an excess above what would be expected based on the general population women. The 95% CIs were calculated using the methods of variance estimates recovery (MOVER) [29]. CIs excluding 0 are indicative of statistical significance at p<0.05. All analyses were performed using Stata version 15.

## Results

### Demographics

Women in the general population (CCHS data) were older than those in the CHIWOS sample: 34.4% of the general population women versus only 12.0% of WLWH were >55 years old. Around three-quarter of general population women were White (75.2%) and the rest were either Black (3.2%), Indigenous (2.4%) or other/multi ethnicities (19.2%). However, the ethnoracial identity of CHIWOS sample were White (41.1%), African/Caribbean/Black (29.4%), Indigenous (22.3%), and other ethnicities (7.2%). The distribution of age and ethnoracial groups for both CHIWOS and CCHS is presented in **S1 Table**.

The mean age of all WLWH at time-point 1 was 42.8 (standard deviation [SD]: 10.6). The majority identified as cisgender/non-transgender women (sex-labeled-at-birth and gender identity congruent) (96%) while the rest identified as transgender women. Almost one-quarter (25.1%) were living with HIV for 5 years or less, 40.2% were living with HIV for 6–14 years, and less than one-third were living with HIV for more than 14 years. Overall, 61.0% were optimally on HIV treatment (i.e., treatment adherence ≥ 95%), 22.0% were sub-optimally on HIV treatment (treatment adherence < 95%), while the rest at time-point 1 of the survey were not engaged in HIV treatment. Among those who were on treatment (either optimally or sub-optimally), 87.0% reported an undetectable viral load (i.e., <50 copies/mL). The history of lifetime injection drug use, sex work involvement, and incarceration was reported by 30.9%, 16.6%, and 36.9% of WLWH, respectively (**S2 Table**).

### Relationship, education, income and source of income

Proportions of indicators of relationship status, education, poverty, and main source of income differed significantly between WLWH and estimates expected based on the age-/ethnoracial-standardized general population. The proportion who were single was higher among WLWH compared with the general population (48.7% vs. 26.6%; SPD 22.1% [95% CI: 18.8, 25.4]), while a lower proportion of WLWH reported being married or in a common-law relationship status than their general population counterparts (32.1% vs. 55.3%; SPD -23.2% [95% CI: -26.7, -19.6]). A lower proportion of WLWH had a university education than the general population (14.1% vs. 27.9%; SDP -13.7% [95% CI: -16.8, -10.6]), whereas a higher proportion had an education level of less than high school (16.1% vs. 12.3%; SPD 3.8% [95% CI: 1.5, 6.1]). More than two-third (70.3%) of WLWH versus less than one-third (28.1%) of women of the general population reported a personal income <$20,000 annually, yielding an SPD 42.2% (95% CI: 39.1, 45.2). A higher proportion of WLWH also reported a household income <$20,000 than the estimate expected in the general population sample (65.3% vs. 10.9%; SPD 54.4% [51.5, 57.3]). Finally, 22.1% of WLWH compared with 69.9% of their counterparts in the general population reported having wages/salaries (i.e., paid jobs) as their main source of income (SPD -47.8% [-50.9, -44.6]), while a high proportion of WLWH (62.2%) reported having an employment insurance/compensation/welfare as their main source of income versus only 9.5% of the general population women (SPD 52.7% [95% CI: 49.5, 55.8]) (**Table 1**).

**Table 1:** Comparing sociodemographic variables of women living with HIV (CHIWOS; 2013–2015) and the general population of women in Canada (CCHS; 2013–2014)*.

	CHIWOS	CCHS estimates		SPD^¥^
		CCHS^£^	AER Std.^†^	
**Relationship status**				
Single	48.7 (46.1, 51.3)^‡^	24.3 (23.7, 24.8)	26.6 (24.6, 28.7)	22.1 (18.8, 25.4)
Married or common-law	32.1 (29.7, 34.6)	58.0 (57.3, 58.7)	55.3 (52.7, 57.9)	-23.2 (-26.7, -19.6)
Separated/divorced/widowed	19.2 (17.2, 21.3)	17.7 (17.2, 18.3)	18.1 (15.8, 20.4)	1.1 (-1.9, 4.2)
**Education**				
Less than high school	16.1 (14.2, 18.1)	15.4 (14.9, 16.0)	12.3 (11.0, 13.6)	3.8 (1.5, 6.1)
High school completed	37.6 (35.1, 40.2)	24.7 (24.0, 25.4)	23.9 (21.9, 25.9)	13.7 (10.5, 16.9)
Diploma/trade/college	32.2 (29.8, 34.7)	30.6 (29.8, 31.3)	35.9 (33.4, 38.4)	-3.7 (-7.1, -0.11)
University degree (≥Bachelor’s degree)	14.1 (12.4, 16.1)	29.3 (28.6, 30.1)	27.9 (25.4, 30.4)	-13.8 (-16.8, -10.6)
**Yearly personal income** ^a^				
<20,000 CAD ^b^	70.3 (67.8, 72.6)	29.1 (28.4, 29.9)	28.1 (26.1, 30.0)	42.2 (39.1, 45.2)
20,000 to <40,000 CAD	17.2 (15.3, 19.3)	24.8 (24.1, 25.5)	24.5 (22.4, 26.7)	-7.3 (-10.1, -4.1)
≥ 40,000 CAD	10.1 (8.7, 11.8)	30.0 (29.2, 30.8)	33.1 (30.4, 35.8)	-23.0 (-0.26.06, -19.55)
Not stated	2.4 (1.7, 3.3)	16.1 (15.5, 16.8)	14.3 (12.3, 16.3)	-11.9 (-14.0, -9.7)
**Yearly household income**				
<20,000 CAD	65.3 (62.8, 67.8)	9.3 (8.9, 9.8)	10.9 (9.5, 12.3)	54.4 (51.5, 57.3)
20,000 to <40,000 CAD	20.6 (18.5, 22.8)	20.4 (19.7, 21.0)	18.9 (16.8, 21.1)	1.7 (-1.29, 4.74)
≥ 40,000 CAD	14.1 (12.3, 16.0)	70.3 (69.6, 71.1)	70.2 (67.8, 72.6)	-56.1 (-59.1, -53.0)
**Main source of income**				
Wages/salaries (paid jobs)	22.1 (20.0, 24.3)	59.2 (58.4, 60.1)	69.9 (67.6, 72.2)	-47.8 (-50.9, -44.6)
Employment insurance/ compensation / welfare	62.2 (59.6, 64.7)	4.8 (4.40, 5.1)	9.5 (7.6, 11.4)	52.7 (49.5, 55.8)
Others (ex. dividends and interest, pension, no income, etc.))	15.0 (13.3, 17.0)	29.4 (28.7, 30.0)	17.0 (15.7, 18.3)	-2.0 (-4.1, 0.4)
Don’t know or not stated	0.70 (0.38, 1.30)	6.6 (6.2, 7.1)	3.6 (2.6, 4.6)	-2.9 (-3.9, -1.7)

* The Canadian HIV Women’s Sexual and Reproductive Health Cohort Study (CHIWOS; N = 1,422) and the Canadian Community Health Survey (CCHS; analytic N = 46,851)

^‡^ Data are % (95% Confidence Intervals (CIs))

^£^ Unstandardized weighted estimates are reported from CCHS

^†^ AER Std.: Age- and ethnoracial-standardized expected estimates from CCHS

^¥^ SPD: standardized prevalence difference (% (95% CIs)), with positive (negative) values indicating higher (lower) prevalence in WLWH in excess of (less than) what would be expected of Canadian women of similar ages/ethnoracial backgrounds

^a^ aged > 17 years old

^b^ Canadian dollar (CAD)

### Food security

Proportions of food sufficiency and food security were substantially higher in WLWH compared with expected estimates from the general population women. A higher proportion of WLWH reported *sometimes* or *often* their household did not have enough to eat over the last 12 months (15.7% vs. 2.6%; SPD13.1% [95% CI: 10.9, 15.7]), and had enough but not always the kinds of food (53.7% vs. 15.3%; SPD 38.4% [95% CI: 34.4, 42.4]). The analysis of the individual items of food security scale showed that a higher proportion of WLWH reported their household sometimes/often “worried that food would run out before you got money to buy more” (65.7% vs. 17.9%), “the food bought didn’t last and there wasn’t any money to get more” (62.9% vs. 14.3%), and “couldn’t afford to eat balanced meals” (62.7% vs. 14.0%). Overall, a higher proportion of WLWH reported experiencing severe (54.1% vs. 10.2%; SPD 43.9% [95% CI: 40.2, 47.5]), moderate (10.3% vs. 5.3%; SPD 5.0% [95% CI: 2.6, 7.6]), and mild (8.2% vs. 5.2%; SPD 3.0% [95% CI: 1.1, 5.1]) food insecurity than the expected values in the general population (**Table 2**).

**Table 2:** Comparing food sufficiency and food security between women living with HIV (CHIWOS; 2013–2015) and the general population of women in Canada (CCHS; 2013–14)*.

	CHIWOS	CCHS estimates		SPD^¥^
		CCHS^£^	AER Std.^†^	
**Food sufficiency**				
Always had enough of the kinds of food	30.5 (27.8, 33.4)^‡^	89.6 (88.9, 90.2)	82.0 (79.2, 84.8)	-51.5 (-55.4, -47.5)
Had enough, but not always the kinds of food	53.7 (50.7, 56.7)	9.1 (8.5, 9.7)	15.3 (12.7, 18.0)	38.4 (34.4, 42.4)
Sometimes or often did not have enough to eat	15.7 (13.7, 18.1)	1.3 (1.1, 1.6)	2.6 (1.7, 3.6)	13.1 (10.9, 15.7)
**Food security items**				
*Item 1) Food run out*				
Never	34.3 (31.5, 37.2)	90.8 (90.2, 91.4)	82.1 (79.5, 84.7)	-47.8 (-51.5, -43.8)
Sometimes/often	65.7 (62.7, 68.5)	9.2 (8.6, 9.8)	17.9 (15.3, 20.5)	47.8 (43.8, 51.5)
*Item II) Food did not last*				
Never	37.1 (34.3, 40.1)	93.3 (92.7, 93.9)	85.7 (83.3, 88.0)	-48.5 (-52.2, -44.7)
Sometimes/often	62.9 (59.9, 65.7)	6.7 (6.1, 7.2)	14.3 (12.0, 16.7)	48.5 (44.7, 52.2)
*Item III) Could not afford for balanced meal*				
Never	37.3 (34.4, 40.3)	92.8 (92.2, 93.3)	86.0 (83.5, 88.4)	-48.6 (-52.4, -44.8)
Sometimes/often	62.7 (59.7, 65.5)	7.2 (6.6, 7.7)	14.0 (11.6, 16.5)	48.6 (44.8, 52.4)
**Overall Food security** ^*a*^				
Food secure	27.4 (24.8, 30.2)	88.6 (88.0, 89.3)	79.3 (76.7, 82.0)	-51.9 (-55.6, -48.0)
Mildly food insecure	8.2 (6.7, 10.0)	4.1 (3.7, 4.5)	5.2 (4.1, 6.4)	3.0 (1.1, 5.1)
Moderately food insecure	10.3 (8.6, 12.3)	2.7 (2.4, 3.0)	5.3 (3.6, 6.9)	5.0 (2.6, 7.6)
Severely food insecure	54.1 (51.0, 57.0)	4.5 (4.0, 5.0)	10.2 (8.1, 12.2)	43.9 (40.2, 47.5)

* CHIWOS-Ontario/Quebec (N = 1,066) and CCHS-Ontario/Quebec (N = 33,704)

^‡^ Data are % (95% Confidence Intervals (CIs))

^£^ Unstandardized weighted estimates are reported from the Canadian Community Health Survey (CCHS)

^†^ AER Std.: Age- and ethnoracial-standardized expected estimates from CCHS

^¥^ SPD: standardized prevalence difference (% (95% CIs)), with positive (negative) values indicating higher (lower) prevalence in WLWH in excess of (less than) what would be expected of Canadian women of similar ages/ethnoracial backgrounds

^a^ The summation of three binary items (0, indicating Never true and 1, indicating sometimes/often true) of the scale produced an index ranging from 0 to 3; 0: food secure, 1: mild food insecurity, 2: moderate food insecurity, and 3: severe food insecurity.

### Social support and discriminations

Analyzing the overall binary measure of perceived social support showed that a higher proportion of WLWH reported poorer social support compared with the general population women adjusted for age and ethnoracial group status (30.3% vs. 2.9%; SPD 27.4% [95% CI: 22.2, 33.0]). WLWH reported experiencing frequent racial discrimination (46.4% vs. 9.6%; SPD 36.8% [95% CI: 31.9, 41.8]) and frequent gender discrimination (54.4% vs. 8.4%; SPD 46.0% [95% CI: 42.6, 51.6]) than the expected values of the general population women (**Table 3**).

**Table 3:** Comparing social support*, and racial and gender discrimination** between women living with HIV (CHIWOS; 2013–2015) and the general population of women in Canada (CCHS; 2013–2014).

	CHIWOS	CCHS estimates		SPD^¥^
		CCHS^£^	AER Std.^†^	
**Perceived social support** ^a^				
Poor	30.3 (25.6, 35.5)^‡^	1.9 (1.5, 2.3)	2.9 (0.7, 5.1)	27.4 (22.2, 33.0)
Good	69.7 (64.5, 74.4)	98.1 (97.7, 98.5)	97.1 (94.9, 99.3)	-27.4 (-33.0, -22.2)
**Race discrimination**				
Never	45.6 (43.0, 48.2)	93.5 (92.2, 94.8)	87.1 (82.2, 92.1)	-41.5 (-47.1, -36.0)
Infrequent	8.0 (6.7, 9.6)	1.1 (0.65, 1.48)	3.3 (0.5, 6.1)	4.7 (1.7, 7.9)
Frequent	46.4 (43.8, 49.0)	5.4 (4.1, 6.6)	9.6 (5.3, 13.8)	36.8 (31.9, 41.8)
**Gender discrimination**				
Never	37.5 (35.0, 40.0)	89.3 (88.2, 90.5)	89.4 (87.0, 91.7)	-51.9 (-55.3, -48.4)
Infrequent	8.2 (6.9, 9.7)	2.6 (2.1, 3.1)	2.2 (1.2, 3.2)	6.0 (4.3, 7.8)
Frequent	54.4 (51.8, 56.9)	8.1 (7.0, 9.0)	8.4 (6.2, 10.6)	46.0 (42.6, 51.6)

* CHIWOS-Quebec (N = 355) and CCHS-Quebec (N = 11,780)

** CHIWOS-all N = 1,422 and CCHS rapid survey (N = 6,936)

^‡^ Data are % (95% Confidence Intervals (CIs))

^£^ Unstandardized weighted estimates are reported from the Canadian Community Health Survey (CCHS)

^†^ AER Std.: Age- and ethnoracial-standardized expected estimates from CCHS

^¥^ SPD: standardized prevalence difference (% (95% CIs)), with positive (negative) values indicating higher (lower) prevalence in WLWH in excess of (less than) what would be expected of Canadian women of similar ages/ethnoracial backgrounds

^a^ The summation of four items, each having four options (0 to 3), produced an index ranging from 0 to 12; with a lower score indicating lower level of social support.

A binary measure was created based on the mid-point threshold score: score mid-point or below (i.e., ≤ 6) indicated poor/low perceived social support, and scores above mid-point (i.e., > 6) indicated better/good perceived social support.

### Overall health status

A higher proportion of WLWH reported poor and fair overall health status than the estimates expected based on the age-/ethnoracial-standardized assumed HIV-negative women. The aggregated proportion of these two options (i.e., fair/poor health condition), indicating a lower level of overall health status, was higher among WLWH than the general population women (24.8% vs. 12.6%; SPD: 12.2% [95% CI: 9.4, 15.0]) (**Table 4**).

**Table 4:** Comparing self-rated overall health status between women living with HIV (CHIWOS; 2013–2015) and the general population of women in Canada (CCHS; 2013–2014)*.

Self-rated health	CHIWOS	CCHS estimates		SPD^¥^
		CCHS^£^	AER Std.^†^	
*A five-category measure*				
Excellent	8.3 (6.9, 9.8)^‡^	20.7 (20.0, 21.4)	21.9 (19.5, 24.2)	-13.6 (-16.3, -10.8)
Very good	26.9 (24.6, 29.3)	37.5 (36.7, 38.3)	35.8 (33.6, 37.9)	-8.9 (-12.0, -5.7)
Good	40.1 (37.5, 42.6)	30.0 (29.1, 30.8)	29.7 (27.3, 32.1)	10.3 (6.8, 13.8)
Fair	19.0 (17.1, 21.2)	8.8 (8.3, 9.2)	8.9 (7.5, 10.2)	10.2 (7.8, 12.7)
Poor	5.7 (4.6, 7.1)	3.0 (2.8, 3.3)	3.7 (2.8, 4.7)	2.0 (0.51, 3.6)
*A binary measure*				
Excellent/v. good/good	75.2 (72.9, 77.4)	88.2 (87.6, 88.7)	87.4 (85.8, 89.0)	-12.2 (-15.0, -9.4)
Fair/poor	24.8 (22.6, 27.1)	11.8 (11.3, 12.3)	12.6 (11.0, 14.2)	12.2 (9.4, 15.0)

* The Canadian HIV Women’s Sexual and Reproductive Health Cohort Study (CHIWOS; N = 1,422) and the Canadian Community Health Survey (CCHS; analytic N = 46,851)

^‡^ Data are % (95% Confidence Intervals (CIs))

^£^ Unstandardized weighted estimates are reported from CCHS

^†^ AER Std.: Age- and ethnoracial-standardized expected estimates from CCHS

^¥^ SPD: standardized prevalence difference (% (95% CIs)), with positive (negative) values indicating higher (lower) prevalence in women living with HIV (WLWH) in excess of (less than) what would be expected of Canadian women of similar ages/ethnoracial backgrounds.

## Discussion

Drawing on data from the largest cohort study of WLWH in Canada, we found that 42.2% and 43.9% of WLWH respectively reported an annual personal income <$20,000—a low income cut-off indicating poverty—and severe food insecurity, in excess of what would be expected of Canadian women of similar ages/ethnoracial backgrounds. Additionally, a higher proportion of WLWH reported experiencing the proxy indicators for social exclusion including poor perceived social support, and racial and gender discriminations compared with what would be expected. The self-rated health, as a proxy but holistic measure of health, was also lower in WLWH. While previous research highlighted the greater socio-structural disadvantages and economic hardships among WLWH, we are not cognizant of previous comparisons between these two populations.

Although this analysis did not permit assessment of whether living with HIV exacerbated inequities in SDoH or whether such inequities increase risk of acquiring HIV or (likely) a mixture of both, the fact that a large proportion of WLWH in Canada are living with multiple and overlapping disadvantages with regard to social and economic participation is unjust and of huge concern. The concentration of financial hardship, food insecurity, and social exclusion–with having the potential to exposure to increased magnitude of chronic and acute stressors, poses a wide range of barriers that negate the ability of individuals to consistently engage in the HIV care/treatment cascade, e.g., retention in care [30] and cART initiation and continuation [12], and further undermine attempts to optimize the treatment outcomes. Recent studies have documented the role of food insecurity, for example, on cART non-adherence and incomplete HIV viral suppression [10, 15]. Such level of risk has also been realized for social exclusion determinants [14] as found notably prevalent in WLWH in the present study. These findings highlight the need for multi-component interventions targeted at SDoH inequity reduction, particularly in those women with an increased risk for treatment interruptions, discontinuation, and non-adherence due to limited socio-structurally resources.

The substantial differences in the study determinants and self-assessed health identified between the two samples would provide evidence on the socio-structural determinants of WLWH to aid with policy development and resource allocation. Given the concern surrounding the growing proportion of WLWH in Canada [18], our findings have implications for evoking calls for gender-specific tailored service, a complex and multidimensional model of care and service delivery as the current care approaches appear to be inadequate to address women's comprehensive needs. The women-centered model of HIV care that has already been envisioned by target population is recommended to be a useful model of care to guiding policy and practice to improve care and health outcomes [31]. Such models of care require to target the persistent health inequalities in women with HIV, relative to either men with HIV [3, 4] or women of the general pupation, through a social-determinants framework, an approach in which a wide range of disciplines contribute to addressing the underlying barriers and reducing health inequities [32]. Socio-structural approach of addressing the fundamental causes of health inequities are imperative to achieve the UNAIDS “90-90-90 targets”—the universal commitments of HIV epidemic elimination by 2030 [33].

This analysis has also significant implications for designing strategies that support WLWH through social service programs, and reinforce social support and resilience with the objective of facilitating women’s access to care, promoting health and wellbeing, health equity, and social justice. Programs supporting social service delivery have important implications, especially now that HIV care has shifted toward chronicity. The provision of transportation supports, financially accessible complementary services, and providing flexible program schedules can facilitate access to care among women with socio-economical disadvantages [34]. The integration of social programs into health service delivery can help address socio-structural adversities and facilitate women's participation in HIV care.

### Strengths and limitations

To our knowledge, this is the first research investigating the inequities with socio-structural determinants of health and the self-rated health between WLWH and assumed HIV-negative women of the general population. However, this study is not without limitations. First, we compared the health determinants among WLWH with the *assumed* HIV-negative women of the general population. However, due to small population estimates of WLWH in Canada—97 per 100,000 females [18]—we believe the inclusion of WLWH in the comparison group would not substantially impact on our findings. Furthermore, the substantial differences identified between the two surveys may be partly due to differences in population structure other than age/ethnoracial group, factors which were not accounted for in standardization. Moreover, self-report data may be prone to social desirability bias, particularly in CCHS data. CHIWOS attempted to mitigate the impact of this bias using trained peer research associates (PRAs), who shared an experience of living with HIV, to administer the surveys. Also, CHIWOS’s non-random sampling design may undermine the generalizability of these findings.

## Conclusion

These findings provide information on the upstream determinants of health their inequalities in WLWH indicating that a high proportion of WLWH in Canada experienced much worse economic hardships, food insecurity, social exclusions as well as poor/fair self-reported health, in excess of what would be expected. These findings support the need for the integration of socio-structural approaches and health equity into practice to address women’s unique needs. These findings also advocate for social service delivery and programming as well as further resource allocation aim to reduce socially constructed, unjust, and avoidable inequalities in health in this population. Addressing these needs when providing individual-tailored HIV care and treatment services will promote the clinical care of a sizable proportion of women with HIV living in poverty. Future research needs to focus on targeted exclusion-reduction interventions, e.g., poverty- and discrimination-reduction strategies, in this population. Future research could also assess the independent and/or clustered impact of these social determinants of health (e.g., race discrimination, gender discrimination) plus other relevant social determinants in the field of HIV such as HIV-related stigma on health outcomes of WLWH. Applying advanced statistical techniques such as decomposition analysis [35]–a technique to assess health inequalities through decomposing the overall inequality in the study outcomes into the inequality in each contributing determinants, and latent class analysis (LCA) [36]–a method to identify the latent class/clusters of individuals who experience the unique adversities with respect to the social determinants, can help researchers better explore the association of these determinants with HIV outcomes. This data on SDoH inequalities can help investigators develop interventions to address disparities experienced by WLWH to improve their health outcomes, and identify mechanisms through which these determinants may reinforce or directly contribute to inequitable vulnerabilities among WLWH.

## Supporting information

S1 Table Age-ethnoracial distributions of both the CHIWOS (2013–2015) cohort of women living with HIV and the CCHS (2013–2014) data of the corresponding general population women in Canada. (DOCX)

S2 Table Characteristics of Women Living with HIV (WLWH)–CHIWOS Time-point 1, 2013–2015 (N = 1,422). (DOCX)
