# Supplementary material for: Social determinants of health and self-rated health status: A comparison between women with HIV and women without HIV from the general population in Canada
Source: PLoS One. 2019 Mar 21;14(3):e0213901. doi: 10.1371/journal.pone.0213901 (PMC6428327; doi:10.1371/journal.pone.0213901)
Supplement: S1 Table — (DOCX) [file pone.0213901.s001.docx]

**S1 Table.** Age-ethnoracial distributions of both the CHIWOS (2013-2015) cohort of women living with HIV and the CCHS (2013-2014) data of the corresponding general population women in Canada.

|  | **CHIWOS estimates  (N=1,422)** | **CCHS estimates (N=46,851)**^a^ | |
| --- | --- | --- | --- |
|  | **%** | **Unstandardized**  **%** | **Standardized**  **%** |
| **Ethnoracial and age groups** |  |  |  |
| White 16-35 (years) | 10.2^b^ | 21.1^c^ | 10.2^d^ |
| 36-45 | 11.4 | 11.2 | 11.4 |
| 46-55 | 12.5 | 13.9 | 12.5 |
| > 55 | 7.0 | 29.0 | 7.0 |
| Black 16-35 | 7.5 | 1.1 | 7.5 |
| 36-45 | 11.5 | 0.7 | 11.5 |
| 46-55 | 7.8 | 0.8 | 7.8 |
| > 55 | 2.6 | 0.7 | 2.6 |
| Indigenous 16-35 | 7.0 | 1.0 | 7.0 |
| 36-55 | 8.4 | 0.4 | 8.4 |
| 36-45 | 5.2 | 0.4 | 5.2 |
| 46-55 | 1.7 | 0.6 | 1.7 |
| Others 16-35 | 1.4 | 7.9 | 1.4 |
| 36-45 | 2.4 | 4.4 | 2.4 |
| 46-55 | 2.6 | 3.1 | 2.6 |
| > 55 | 0.8 | 3.7 | 0.8 |

^a^ Out of 128,310 respondents, 46,851 (36.5%) were the corresponding general population women for the current study: women aged 16+ residing in three provinces of BC, ON, QC.

^b^ Data are presented as percentages.

^d^ Chi Square test showed a significant difference between the two samples of CHIWOS and unstandardized CCHS (P-value < 0.001)

^c^ Standardization made the two study populations of CHIWOS and CCHS identical with regard to the distribution of age and ethnoracial group.
