# Supplementary material for: Social determinants of health and self-rated health status: A comparison between women with HIV and women without HIV from the general population in Canada
Source: PLoS One. 2019 Mar 21;14(3):e0213901. doi: 10.1371/journal.pone.0213901 (PMC6428327; doi:10.1371/journal.pone.0213901)
Supplement: S2 Table — (DOCX) [file pone.0213901.s002.docx]

**S2 Table. Characteristics of Women Living with HIV (WLWH) –** **the baseline survey of the Canadian HIV Women’s Sexual and Reproductive Health Cohort Study (CHIWOS), 2013-2015**

| Variables | n (%) or  mean [SD] |
| --- | --- |
| **Age,** year (mean [SD]) | 42.8 [10.6] |
| **Age groups** (years) (N = 1,422) |  |
| 16-35 | 372 (26.2) |
| 36-45 | 479 (33.7) |
| 46-55 | 400 (28.1) |
| > 55 | 171 (12.0) |
| **Ethno-racial group** (N = 1,422) |  |
| White | 584 (41.1) |
| African/Caribbean/Black | 418 (29.4) |
| Indigenous | 318 (22.3) |
| Other | 102 (7.2) |
| **Study province** (N = 1,422) |  |
| Ontario | 717 (50.4) |
| British Columbia | 356 (25.0) |
| Quebec | 349 (24.6) |
| **Years living with HIV** (N = 1,374) |  |
| < 6 years | 345 (25.1) |
| 6-14 years | 552 (40.2) |
| > 14 years | 477 (34.7) |
| **Taking treatment** (N = 1,415) |  |
| Yes, optimal (≥ 95%) | 863 (61.0) |
| Yes, suboptimal (< 95%) | 312 (22.0) |
| Not engaged in treatment | 240 (17.0) |
| **Undetectable (, 50 copies/mL) viral load among WLWH on treatment** (N=1,170) | 1018 (87.0) |
| **History of injection drug use** (N = 1,421) | 439 (30.9%) |
| **History of sex work involvement** (N=1,321) | 219 (16.6) |
| **History of incarceration** (N=1,420) | 524 (36.9) |
